# COVID-19 Vaccination Intentions, Concerns, and Facilitators Among US Parents of Children Ages 6 Months Through 4 Years

**DOI:** 10.1001/jamanetworkopen.2022.27437

**Published:** 2022-08-03

**Authors:** Aaron M. Scherer, Courtney A. Gidengil, Amber M. Gedlinske, Andrew M. Parker, Natoshia M. Askelson, Kate R. Woodworth, Christine A. Petersen, Megan C. Lindley

**Affiliations:** 1 Department of Internal Medicine, University of Iowa, Iowa City; 2 RAND Corporation, Boston, Massachusetts; 3 Division of Infectious Diseases, Boston Children’s Hospital and Harvard Medical School, Boston, Massachusetts; 4 RAND Corporation, Piitsburgh, Pennsylvania; 5 Department of Community and Behavioral Health, University of Iowa, Iowa City; 6 Centers for Disease Control and Prevention COVID-19 Response, Atlanta, Georgia; 7 Department of Epidemiology, University of Iowa, Iowa City

## Abstract

**Importance:**

Children aged 6 months through 4 years have become eligible for COVID-19 vaccination, but little is known about parental intentions regarding, concerns about, or facilitators to COVID-19 vaccination for this age group.

**Objectives:**

To evaluate parental intentions, concerns, and facilitators for COVID-19 vaccination for children aged 6 months through 4 years and to help inform the US Centers for Disease Control and Prevention Advisory Committee on Immunization Practices’ deliberations and recommendations for COVID-19 vaccination for children aged 6 months through 4 years.

**Design, Setting, and Participants:**

This cross-sectional study fielded an online survey from February 2 to 10, 2022, among a nonprobability sample of US parents of children aged 6 months through 4 years who were recruited through Qualtrics using quota-based sampling for respondent gender, race and ethnicity, and child age group.

**Main Outcomes and Measures:**

COVID-19 vaccination intentions, time to COVID-19 vaccination, COVID-19 vaccination concerns and facilitators, and trusted COVID-19 vaccination locations for children aged 6 months through 4 years.

**Results:**

The final weighted sample of 2031 participants (73.5% participation rate) had more respondents who identified as male (985; weighted percentage, 54.8%) or White (696; weighted percentage, 66.2%), were aged 25 to 49 years (1628; weighted percentage, 85.6%), had at least a bachelor’s degree (711; weighted percentage, 40.0%), lived in a metropolitan area (1743; weighted percentage, 82.9%) or the South (961; weighted percentage, 43.4%), or received at least 1 dose of a COVID-19 vaccine (1205; weighted percentage, 59.8%). Half of respondents (645; weighted percentage, 45.6%) indicated that they “definitely” or “probably” will vaccinate their child aged 6 months through 4 years once they became eligible. However, only one-fifth (396; weighted percentage, 19.0%) indicated they would get a COVID-19 vaccine for their child in this age group within 3 months of them becoming eligible for vaccination. Vaccine safety and efficacy were parents’ top concerns, and receiving more information about safety and efficacy were the top facilitators to COVID-19 vaccination for this age group. A doctor’s office or clinic and local pharmacy were the most trusted COVID-19 vaccination locations for this age group.

**Conclusions and Relevance:**

These results suggest that only a minority of parents of children in this age group are eager to vaccinate their children within the first few months of eligibility, with widespread concerns about COVID-19 vaccination for this age group. Thus, considerable efforts to increase parental COVID-19 vaccine confidence for children aged 6 months through 4 years may be needed to maximize COVID-19 vaccination for this age group in the United States.

## Key Points

**Question:**

What are parents’ intentions, concerns, and facilitators to COVID-19 vaccination for their children aged 6 months through 4 years?

**Findings:**

In this cross-sectional study of 2031 US adults with children aged 6 months through 4 years, half indicated they intended to get their child a COVID-19 vaccine at some point, but only one-fifth intended to do so within 3 months of the child’s eligibility. The top concerns about and facilitators to COVID-19 vaccination for this age group related to COVID-19 vaccination safety and efficacy.

**Meaning:**

These findings suggest that considerable efforts to increase parental COVID-19 vaccine confidence for children aged 6 months through 4 years may be needed to maximize COVID-19 vaccination for this age group.

## Teaser

This cross-sectional study evaluates whether parents of children aged 6 months through 4 years intend to vaccinate their children against COVID-19 and what concerns or facilitators are associated with the decision to vaccinate this age group.

## Introduction

COVID-19 hospitalizations among children younger than 5 years were 5 times higher during Omicron’s peak than Delta’s.^1^ On June 17, 2022, the US Food and Drug Administration (FDA) extended the Emergency Use Authorizations for the BNT162b2 (Pfizer-BioNTech) and mRNA-1273 (Moderna) COVID-19 vaccines to children aged 6 months through 4 years.^2^ Despite the long wait for this age group to become eligible for COVID-19 vaccination, there have been few peer-reviewed studies that have assessed intentions of parents of children younger than 5 years to get a COVID-19 vaccine for their child in this age group once they become eligible. However, these surveys were fielded in 2021 (February to March^3,4^ and September to October^5^), and only 1 study^3^ examined parents of children younger than 5 years specifically, making their utility for anticipating factors associated with COVID-19 vaccine uptake among children in this age group less than ideal.

The current survey was commissioned by the US Centers for Disease Control and Prevention (CDC) COVID-19 Response to the Healthcare and Public Perceptions of Immunizations (HaPPI) Survey Collaborative to rapidly assess factors that might affect COVID-19 vaccine update for children aged 6 months through 4 years once they became eligible for vaccination. The purpose of this survey was to help inform the Advisory Committee on Immunization Practices’ (ACIP) deliberations and recommendations for COVID-19 vaccination for this age group,^6^ originally scheduled to occur at their meeting on February 23 to 24, 2022, which was delayed until their meeting on June 17 to 18, 2022.

## Methods

### Recruitment

We administered an internet-based survey from February 2 to 10, 2022, to a national, nonprobability sample of US parents of children ages 6 months through 4 years recruited through Qualtrics.^7^ Sampling quotas were used to reduce potential sampling bias. The quotas included child age group (6-23 months and 2-4 years; 1:1 ratio), respondent gender (female and male; 1:1 ratio), and the 3 largest racial and ethnic groups in the United States (Hispanic, non-Hispanic Black, and non-Hispanic White; 1:1:1 ratio) and were tracked via responses to survey items. A minimum sample size of 1901 would be necessary to detect a small (f = 0.10) effect size for an analysis of variance with 6 groups with 80% power and α = .007. The Statistical Analysis has more information regarding the adjusted *P* value rationale.

Inclusion criteria included (1) currently living in the United States; (2) having regular responsibility for a child aged 6 months through 4 years living at home with the respondent; (3) identifying as Black, Hispanic, or White; and (4) after providing consent, indicating (via a survey item) that they would provide their best answers to each survey question. This activity was reviewed and approved by the University of Iowa institutional review board and the CDC and was conducted in a way consistent with applicable federal law and CDC policy. We used the Strengthening the Reporting of Observational Studies in Epidemiology (STROBE) reporting recommendations for this cross-sectional study.

### Procedure

Potentially eligible panelists were identified via the panel’s proprietary algorithm and sent a survey link. Those who clicked on the link were directed to a page that described the study, followed by a screen with a survey item to provide consent and another item to indicate commitment to providing their best answers to each survey question. Panelists who did not consent to participate or agree to provide quality responses were redirected back to the panel website.

Panelists who consented and agreed to provide quality responses then answered screener questions to assess their eligibility for the study, described previously. Respondents with at least 1 child in both age groups (6-23 months; 2-4 years) were randomized to 1 of the 2 age groups. Respondents with more than 1 child in an age group were instructed to think about the child with the most recent birthday for their responses. Respondents also provided demographic information before reading a short introduction to the main part of the survey that highlighted that while children ages 6 months through 4 years were currently ineligible for COVID-19 vaccination, the Emergency Use Authorizations for the COVID-19 vaccines available in the United States were expected to be expanded to this age group in the near future.

### Measures

#### COVID-19 Vaccination for Children Aged 6 Months Through 4 Years

Primary measures were parents’ COVID-19 vaccination intentions for their child (“How likely are you to get a COVID-19 vaccine for your [age] child, if they become eligible for vaccination?”) and time-to-vaccination intentions (“How long do you think you will wait before getting a COVID-19 vaccine for your [age] child if they became eligible for vaccination?”). All respondents completed this latter item, including those reporting they “definitely will not” get a COVID-19 vaccine for their child, given that respondents may have responded to the prior question based on what they plan to do immediately after children aged 6 months through 4 years become eligible for COVID-19 vaccination rather than doing so at any point in the future.

Secondary measures included concerns, facilitators, and vaccination location preferences for their child aged 6 months through 4 years receiving a COVID-19 vaccine. Respondents who indicated that they “definitely will” get a COVID-19 vaccine for their child did not complete the parental concerns measure. Table 1 presents the full list of response options to the 5 COVID-19 vaccination items.

**Table 1.** Unweighted and Weighted Results for Measures Related to COVID-19 Vaccination Among 2031 Parents of Children Aged 6 Months Through 4 Years^a^

Measure	Unweighted, No. (%)	Weighted, % (95% CI)
**COVID-19 vaccination intention for child aged 6 mo through 4 y**		
Definitely will	521 (25.7)	27.2 (25.0-29.6)
Probably will	224 (11.0)	18.4 (16.5-20.5)
Not sure	323 (15.9)	15.6 (13.8-17.6)
Probably will not	392 (19.3)	11.6 (10.0-13.4)
Definitely will not	571 (28.1)	27.2 (24.9-29.6)
**Time-to–COVID-19 vaccination intention for child aged 6 mo through 4 y**		
<3 mo	396 (19.5)	19.0 (17.1-21.2)
3-6 mo	430 (21.2)	20.3 (18.3-22.5)
>6 mo	518 (25.5)	27.0 (24.7-29.4)
Do not know	687 (33.8)	33.7 (31.3-36.2)
**COVID-19 vaccination concerns for child aged 6 mo through 4 y** ^b^ ^,^ ^c^		
I am concerned about possible long-term side effects of a COVID-19 vaccine	697 (47.7)	51.6 (48.5-54.7)
I am concerned about possible short-term side effects of a COVID-19 vaccine	550 (37.7)	40.8 (37.7-43.8)
I plan to wait and see if it is safe and may get it for my child later	494 (33.8)	34.0 (31.1-36.9)
I am concerned about my child experiencing myocarditis or pericarditis (inflammation in or around the heart) to a COVID-19 vaccine	479 (32.8)	34.3 (31.4-37.3)
I am concerned about my child having an allergic reaction to a COVID-19 vaccine	458 (31.4)	31.4 (28.6-34.3)
I don’t trust COVID-19 vaccines	455 (31.2)	32.5 (29.6-35.4)
I don’t know if a COVID-19 vaccine will work	322 (22.1)	23.6 (20.9-26.2)
I don’t believe my child needs a COVID-19 vaccine	303 (20.8)	23.5 (20.8-26.2)
I don’t think COVID-19 is that big of a threat for my child	222 (15.1)	18.6 (16.0-21.1)
My child doesn’t like needles	137 (9.4)	9.1 (7.4-10.9)
I have faith-based objections to my child receiving a COVID-19 vaccine	131 (9.0)	8.1 (6.5-9.8)
I think other people need it more than my child does right now	126 (8.6)	7.6 (6.0-9.1)
I am concerned about the cost of a COVID-19 vaccine	78 (5.3)	4.4 (3.2-5.6)
Other people in my community are choosing not to get their children vaccinated	74 (5.1)	4.9 (3.6-6.3)
There are obstacles that may prevent me from getting a COVID-19 vaccine for my child	66 (4.5)	3.7 (2.6-4.7)
None of these	39 (2.7)	2.4 (1.5-3.3)
Other	34 (2.3)	2.9 (1.8-4.0)
**Facilitators for COVID-19 vaccination of child aged 6 mo through 4 y^c^**		
More information showing COVID-19 vaccines are safe in children was available	569 (28.0)	30.5 (28.0-32.9)
More information showing COVID-19 vaccines are effective in children was available	536 (26.4)	28.3 (25.9-30.7)
Cases of COVID-19 in children or young adults got more severe	422 (20.8)	21.3 (19.2-23.5)
One or more COVID-19 vaccines received full FDA approval for children aged 6 mo to 4 y	415 (20.4)	21.3 (19.2-23.5)
It would reduce the spread of COVID-19 in my child’s community	382 (18.8)	9.6 (8.1-11.2)
It would prevent my child from spreading COVID-19 to family and friends	379 (18.7)	18.9 (16.8-21.0)
None of these	355 (17.5)	18.1 (16.1-20.2)
It was recommended for my child by a health care provider	350 (17.2)	18.4 (16.4-20.5)
We keep seeing new variants, like the Omicron variant	341 (16.8)	17.0 (15.0-18.9)
A big increase in COVID-19 cases in my area	319 (15.7)	15.5 (13.6-17.4)
It would allow my child to resume or do more social activities	314 (15.5)	15.4 (13.5-17.3)
The vaccine could be sprayed in the nose or swallowed	302 (14.9)	15.6 (13.7-17.6)
My child’s daycare or other childcare facility required it	270 (13.3)	13.4 (11.6-15.2)
Someone I personally know became seriously ill or died from COVID-19	238 (11.7)	11.5 (9.9-13.2)
It would allow my child to travel	224 (11.0)	10.7 (9.1-12.3)
It would mean my child would not have to quarantine if they were exposed to COVID-19	199 (9.8)	10.3 (8.7-11.9)
My child’s extracurricular activities (eg, sports team) required it	196 (9.7)	9.1 (7.7-10.6)
I saw people in my community getting their children vaccinated against COVID-19	193 (9.5)	9.6 (8.1-11.2)
It was recommended for my child by a family member or friend	149 (7.3)	6.4 (5.2-7.7)
**Parental COVID-19 vaccination location preferences for child aged 6 mo through 4 y^c^**		
Your child’s regular doctor’s office or clinic	1282 (63.1)	64.1 (61.6-66.6)
A doctor’s office or clinic, but not your child’s usual one	436 (23.4)	24.5 (22.3-26.9)
A local pharmacy	482 (23.7)	22.8 (20.7-25.1)
None of these		20.7 (18.6-22.9)
A temporary indoor COVID-19 vaccine clinic	335 (16.5)	16.9 (15.0-19.0)
Your child’s daycare or other childcare facility with you or another caregiver present	311 (15.3)	15.0 (13.2-16.9)
A temporary outdoor COVID-19 vaccine clinic (eg, a drive through)	256 (12.6)	12.9 (11.2-14.7)
Your child’s daycare or other childcare facility without you or another caregiver present	168 (8.3)	7.5 (6.2-8.9)

^a^ Survey weights created using population-level rates of gender and race and ethnicity.

^b^ Item completed by 1460 respondents selecting a response other than “Definitely will get a vaccine” for their child.

^c^ Respondents could select any option that applied, resulting in column percent total greater than 100%.

#### Respondent Characteristics

Respondents indicated their gender, race, Hispanic or Latino/a ethnicity, highest level of educational attainment, US state and zip code of primary residence, COVID-19 vaccination status, and the age of their child.

### Statistical Analysis

Descriptive statistics and χ^2^ analyses to test for group differences were conducted using Stata version 14.2 (StataCorp). We conducted analyses without and with a weighting variable (based on national distributions of gender^8^ and race and ethnicity^9^ of US parents of children aged 6 months through 4 years). Both sets of descriptive statistics are reported in Tables 1 and 2, but we report weighted results in the Results section. To account for multiple comparisons, *P* ≤ .007 was considered statistically significant, and all tests were 2-tailed. There were no missing data for the variables included in the current analyses.

**Table 2.** Unweighted and Weighted Characteristics of 2031 Respondents^a^

Sample characteristic	Unweighted, No. (%)	Weighted, % (95% CI)
Parent gender		
Male	985 (48.5)	54.8 (52.2-57.4)
Female	1046 (51.5)	45.2 (42.6-47.8)
Parent age, y		
18-24	360 (17.8)	12.5 (11.0-14.1)
25-49	1628 (80.7)	85.6 (83.9-87.2)
50-64	30 (1.5)	1.9 (1.3-2.8)
≥65	NA	NA
Parent race and ethnicity		
Hispanic	669 (32.9)	20.2 (18.7-21.9)
Non-Hispanic Black	666 (32.8)	13.6 (12.5-14.7)
Non-Hispanic White	696 (34.3)	66.2 (64.1-68.2)
Parent education		
≤High school degree	676 (33.3)	30.1 (27.8-32.5)
Some college or trade school certificate	642 (31.6)	29.9 (27.6-32.4)
≥Bachelor’s degree	711 (35.0)	40.0 (37.4-42.6)
Metropolitan status^b^		
Metropolitan area	1743 (85.4)	82.9 (79.8-85.6)
Nonmetropolitan area	297 (14.6)	17.1 (14.4-20.2)
Region		
Northeast	303 (14.9)	15.6 (13.7-17.6)
Midwest	425 (20.8)	23.8 (21.6-26.2)
South	47.1 (961)	43.4 (40.8-46.0)
West	17.1 (350)	17.2 (15.3-19.2)
Parent vaccination status		
≥1 COVID-19 vaccine dose	1205 (59.3)	59.8 (57.2-62.3)
Unvaccinated or unknown status	826 (40.7)	40.2 (37.7-42.8)
Child age		
6-23 mo	1010 (49.7)	50.0 (47.3-52.6)
2-4 y	1021 (50.3)	50.0 (47.4-52.7)

Abbreviation: NA, not applicable.

^a^ Survey weights created using population-level rates of gender and race and ethnicity.

^b^ Categorized by Rural-Urban Commuting Area (RUCA) code.

## Results

### Sample Characteristics

Of the 2765 respondents who started the survey, 561 closed the web browser before reaching the end, 156 completed the survey too quickly (one-third of the median completion time), and 17 were excluded for describing their gender as “transgender” or “none of these,” resulting in group sizes being too small for statistical analyses. The final sample was 2031 respondents (73.5% participation rate).

The final weighted sample had more respondents who identified as male (985; weighted percentage, 54.8%) or White (696; weighted percentage, 66.2%), were aged 25 to 49 years (1628; weighted percentage, 85.6%), had a bachelor’s degree or higher (711; weighted percentage, 40.0%), and lived in a metropolitan area (1743; weighted percentage, 82.9%) or the South (961; weighted percentage, 43.4%) (Table 2). Respondents were evenly split between answering for a child aged 6 to 23 months or a child aged 2 to 4 years, and 1205 (weighted percentage, 59.8%) reported receiving at least 1 dose of a COVID-19 vaccine.

### COVID-19 Vaccination Intentions for Children Aged 6 Months Through 4 Years

**Figure 1.**
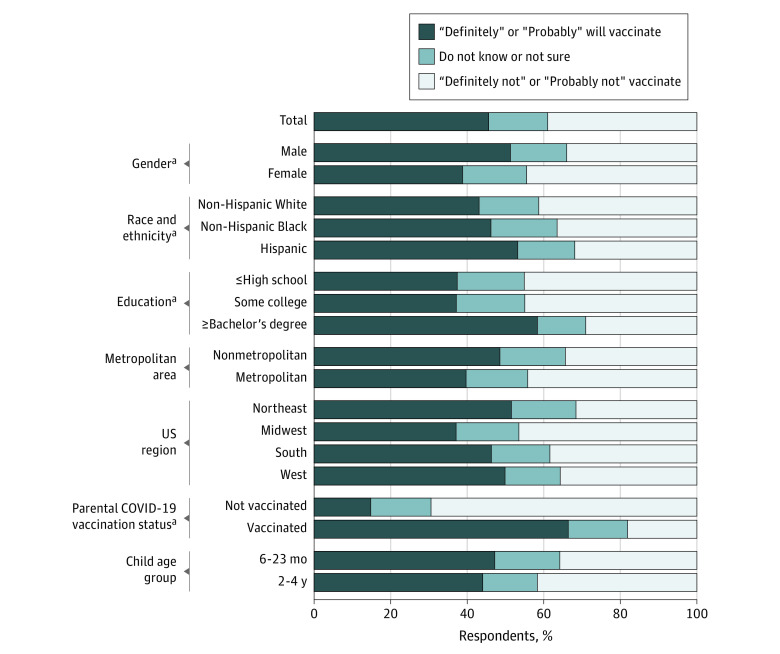
Weighted Differences Among 2031 US Parents Regarding COVID-19 Vaccination Intentions for Children Aged 6 Months Through 4 Years by Demographic Group, February 2022 ^a^*P* ≤ .007.

Nearly half (645 [45.6%]) of respondents reported they “definitely” or “probably” will vaccinate their child when eligible (Table 1). Vaccination intentions were lower for respondents who identified as female (38.8%; 95% CI, 35.4%-42.4%) vs male (51.3%; 95% CI, 47.6%-55.0%; χ^2^_2_ = 32.54; *P* < .001), White (43.2%; 95% CI, 39.6%-47.0%) vs Hispanic (53.2%; 95% CI, 49.4%-56.9%; χ^2^_4_ = 14.88; *P* < .001), had less education (≤high school degree: 37.4%; 95% CI, 33.1%-41.9%; some college or trade school certificate: 37.2%; 95% CI, 32.8%-41.8%) vs a bachelor’s degree or more (58.3%; 95% CI, 54.0%-62.5%; χ^2^_4_ = 87.47; *P* < .001), or were unvaccinated against COVID-19 (14.8%; 95% CI, 12.2%-17.9%) vs at least partially vaccinated (66.4%; 95% CI, 63.1%-69.6%; χ^2^_2_ = 616.39; *P* < .001) (Figure 1). Intentions did not significantly differ by metropolitan status (χ^2^_2_ = 6.25; *P* = .14), US Region (χ^2^_6_ = 25.03; *P* = .01), or child age group (χ^2^_2_ = 8.05; *P* = .06).

**Figure 2.**
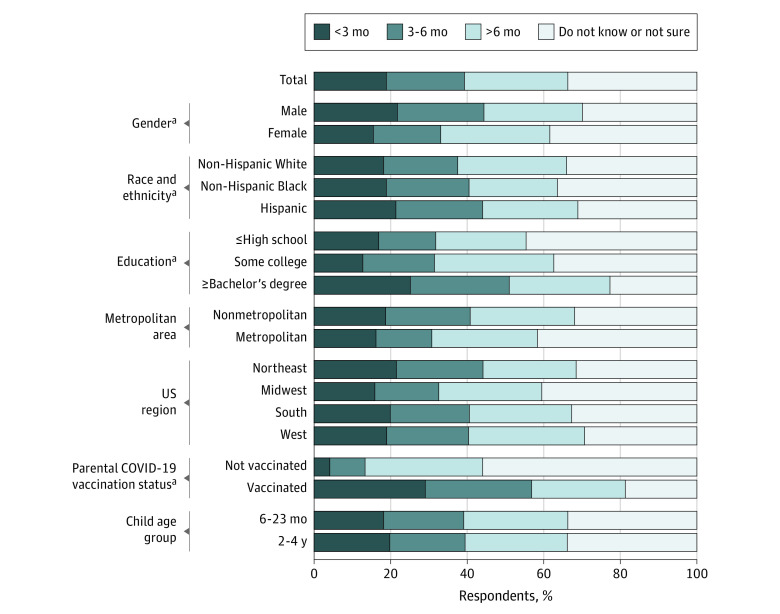
Weighted Differences Among 2031 US Parents in Time-to–COVID-19 Vaccination for Children Aged 6 Months Through 4 Years by Demographic Group, February 2022 ^a^*P* ≤ .007.

One-fifth (19.0%; 95% CI, 17.1%-21.2%) of respondents would vaccinate their child within 3 months of eligibility, 47.3% (95% CI, 42.9%-51.7%) would wait longer, and 33.7% (95% CI, 31.3%-36.2%) did not know when or if they would vaccinate their child (Figure 2). There was a significantly lower percentage of respondents who planned on vaccinating their child aged 6 months through 4 years within 3 months of becoming eligible among those who identified as female (15.6%; 95% CI, 13.2%-18.4%) vs male (21.8%; 95% CI, 18.9%-25.1%; χ^2^_3_ = 29.05; *P* < .001), had less education (≤high school degree: 16.9%; 95% CI, 13.8%-20.6%; some college or trade school certificate: 12.9%; 95% CI, 10.2%-16.33%) vs a bachelor’s degree or more (25.2%; 95% CI, 21.7%-29.1%; χ^2^_6_ = 110.83; *P* < .001), or were unvaccinated against COVID-19 (4.1%; 95% CI, 2.8%-5.9%) vs at least partially vaccinated (29.1%; 95% CI, 26.1%-32.3%; χ^2^_3_ = 451.78; *P* < .001) (Figure 2). There were no significant time-to–COVID-19 vaccination differences by race and ethnicity (χ^2^_6_ = 8.45; *P* = .09), metropolitan status (χ^2^_3_ = 8.26; *P* = .15), US region (χ^2^_9_ = 19.34; *P* = .16), or child age group (χ^2^_3_ = 1.11; *P* = .86).

The time-to–COVID-19 vaccination results did not meaningfully change when 521 respondents who indicated that they “definitely will not” vaccinate their child aged 6 months through 4 years were excluded. Of the 1510 respondents indicating some possibility of COVID-19 vaccination for their child aged 6 months through 4 years, approximately one-quarter (25.7%; 95% CI, 23.13%-28.5%) would vaccinate within 3 months of them becoming eligible, 50.3% (95% CI, 45.1%-55.8%) would wait longer, and 24.0% (95% CI, 21.4%-26.7%) did not know when or if they would vaccinate their child.

### Concerns and Facilitators Regarding COVID-19 Vaccination for Children Aged 6 Months Through 4 Years

The most commonly selected concerns about COVID-19 vaccination for the respondents’ child aged 6 months through 4 years pertained to vaccine safety (Table 1). The 1839 respondents who were shown the concern item (ie, those who did not select “definitely will vaccinate”) indicated that they had at least 1 concern about their child receiving a COVID-19 vaccine. More information on COVID-19 vaccine safety (30.5% [95% CI, 28.0%-32.9%]) or efficacy (28.3% [95% CI, 25.9%-30.7%]) for this age group were the most-selected facilitators, followed by full FDA approval of a COVID-19 vaccine for this age group or COVID-19 cases becoming more severe (both 21.3%) (Table 1).

### Trusted Locations for Children Aged 6 Months Through 4 Years to Get a COVID-19 Vaccine

Approximately two-thirds of respondents indicated that they would feel comfortable with their child aged 6 months through 4 years being vaccinated at their child’s regular doctor’s office or clinic (64.1% [95% CI, 61.6%-66.6%]), while approximately one-quarter reported being comfortable with their child receiving a COVID-19 vaccine at a different doctor’s office or clinic (24.5% [95% CI, 22.3%-26.9%]) or a local pharmacy (22.8% [95% CI, 20.7%-25.1%]) (Table 1).

## Discussion

Almost half of parents of children aged 6 months through 4 years reported that they “definitely” or “probably” will vaccinate their child once they became eligible for COVID-19 vaccination. However, only one-fifth of respondents intended to get their child in this age range a COVID-19 vaccine within 3 months of the child becoming eligible for vaccination. The overall COVID-19 vaccination intentions for children in this age group were lower for respondents who identified as female or White, had lower education, or were unvaccinated themselves.

Our results highlight the persistence of safety and efficacy concerns for COVID-19 vaccination. Excluding respondents who said they “definitely will” get their child aged 6 months through 4 years a COVID-19 vaccine once they become eligible, every respondent indicated that they had at least 1 concern about their child getting a COVID-19 vaccine. This result highlights that widespread hesitancy around COVID-19 vaccination for children younger than 5 years exists, even among those who are inclined to vaccinate their child in this age group.

Although there have been some published studies examining parents’ COVID-19 vaccination intentions for US children younger than 5 years,^4,5,6^ the data for these studies were collected in spring and fall 2021 and most did not look at younger-than-5-years age group specifically but at children aged as old as 12 years, making it challenging to use this data to estimate COVID-19 vaccine uptake among children younger than 5 years now that they are eligible for vaccination. In contrast, our survey was fielded with parents of children in this age group specifically, just weeks before these parents originally thought their child might become eligible for COVID-19 vaccination in February 2022.

### Limitations

This study has limitations. A key limitation was the use of nonprobability sampling, which introduces the potential for sampling bias. While probability-based sampling is ideal for getting precise population estimates, it is also time- and resource-intensive. Thus, probability-based sampling was not a feasible option for our survey given the 2-week deadline the HaPPI Survey Collaborative was given to provide the CDC with the requested information. To mitigate potential sampling bias, we used quota-sampling to ensure sufficient numbers of respondents for respondent race and ethnicity and roughly equal group sizes for respondent gender, race and ethnicity, child age group during recruitment. Additionally, we conducted statistical analyses using a weighting variable created from population distributions of gender and race and ethnicity for parents of children younger than 5 years.^8,9^

Another key limitation was the exclusion of parents who did not identify as Black, Hispanic, or White. The CDC decision to only recruit Black, Hispanic, and White respondents was to ensure that there were a sufficient number of respondents from the 3 largest racial and ethnic groups in the United States for to be able to make meaningful group comparisons.

## Conclusions

Despite the long wait for children aged 6 months through 4 years to become eligible for COVID-19 vaccination, our results suggest that only a minority of parents of children in this age group are eager to vaccinate them now that they are eligible. The hesitancy of parents of children in this age group to get their child a COVID-19 vaccine may reflect the fact that even parents who reported that they “probably will” vaccinate their child have at least 1 major concern about their child receiving a COVID-19 vaccine. Taken together, our results suggest that considerable efforts to increase parental COVID-19 vaccine confidence for children aged 6 months through 4 years may be needed in the United States to maximize COVID-19 vaccination for this age group.

## References

[1] Marks KJ, Whitaker M, Agathis NT, ; COVID-NET Surveillance Team. Hospitalization of infants and children aged 0-4 years with laboratory-confirmed COVID-19—COVID-NET, 14 States, March 2020-February 2022. MMWR Morb Mortal Wkly Rep. 2022;71(11):429-436. doi:10.15585/mmwr.mm7111e2 35298458 PMC8942304

[2] Federal Drug Administration. Coronavirus (COVID-19) update: FDA authorizes Moderna and Pfizer-BioNTech COVID-19 vaccines for children down to 6 months of age. June 17, 2022. Accessed June 22, 2022. https://www.fda.gov/news-events/press-announcements/coronavirus-covid-19-update-fda-authorizes-moderna-and-pfizer-biontech-covid-19-vaccines-children

[3] Szilagyi PG, Shah MD, Delgado JR, . Parents’ intentions and perceptions about COVID-19 vaccination for their children: results from a national survey. Pediatrics. 2021;148(4):e2021052335. doi:10.1542/peds.2021-052335 34344800 PMC10116994

[4] Teasdale CA, Borrell LN, Kimball S, . Plans to vaccinate children for COVID-19: a survey of US parents. J Pediatr. 2021;237:292-297. doi:10.1101/2021.05.12.21256874 34284035 PMC8286233

[5] Willis DE, Schootman M, Shah SK, . Parent/guardian intentions to vaccinate children against COVID-19 in the United States. Hum Vaccines Immunother. Published online May 4, 2022. doi:10.1080/21645515.2022.2071078 PMC9302502 35506876

[6] US Centers for Disease Control and Prevention. ACIP presentation slides: June 18, 2022, meeting. Accessed June 24, 2022. https://www.cdc.gov/vaccines/acip/meetings/slides-2022-06-18.html

[7] Qualtrics. What is a research panel? Accessed January 6, 2022. https://www.qualtrics.com/experience-management/research/research-panels-samples/

[8] United States Census Bureau. Table S0101: 2019 American Community Survey 1-year estimates—age and sex. Accessed June 23, 2022. https://data.census.gov/cedsci/table?q=Age%20and%20Sex&tid=ACSST1Y2019.S0101

[9] United States Census Bureau. Table B03002: 2019 American Community Survey 1-year estimates—Hispanic or Latino origin by race. Accessed June 23, 2022. https://data.census.gov/cedsci/table?t=Hispanic%20or%20Latino&tid=ACSDT1Y2019.B03002&hidePreview=true

